# Cross‐species signaling pathways analysis inspire animal model selections for drug screening and target prediction in vascular aging diseases

**DOI:** 10.1111/eva.13708

**Published:** 2024-06-10

**Authors:** Fei Sun, Xingxing Chen, Shuqing Zhang, Haihong Jiang, Tianhong Chen, Tongying Xing, Xueyi Li, Rabia Sultan, Zhimin Wang, Jia Jia

**Affiliations:** ^1^ School of Life Sciences Shanghai University Shanghai China; ^2^ Sino‐Swiss Institute of Advanced Technology, School of Micro‐Electronics Shanghai University Shanghai China; ^3^ Shanghai‐MOST Key Laboratory of Health and Disease Genomics Shanghai Institute for Biomedical and Pharmaceutical Technologies Shanghai China

**Keywords:** animal model, cross‐species signaling pathway analysis, drugs in research, single‐cell RNA‐seq, vascular aging

## Abstract

Age is a significant contributing factor to the occurrence and progression of cardiovascular disease (CVD). Pharmacological treatment can effectively alleviate CVD symptoms caused by aging. However, 90% of the drugs have failed in clinics because of the loss of drug effects or the occurrence of the side effects. One of the reasons is the disparity between animal models used and the actual physiological levels in humans. Therefore, we integrated multiple datasets from single‐cell and bulk‐seq RNA‐sequencing data in rats, monkeys, and humans to identify genes and pathways with consistent/differential expression patterns across these three species. An approach called “Cross‐species signaling pathway analysis” was developed to select suitable animal models for drug screening. The effectiveness of this method was validated through the analysis of the pharmacological predictions of four known anti‐vascular aging drugs used in animal/clinical experiments. The effectiveness of drugs was consistently observed between the models and clinics when they targeted pathways with the same trend in our analysis. However, drugs might have exhibited adverse effects if they targeted pathways with opposite trends between the models and the clinics. Additionally, through our approach, we discovered four targets for anti‐vascular aging drugs, which were consistent with their pharmaceutical effects in literatures, showing the value of this approach. In the end, software was established to facilitate the use of “Cross‐species signaling pathway analysis.” In sum, our study suggests utilizing bioinformatics analysis based on disease characteristics can help in choosing more appropriate animal models.

## 1 INTRODUCTION

Cardiovascular disease (CVD) is the leading cause of mortality and morbidity in developed countries (Benjamin et al., 2017). Aging is a major risk factor for cardiovascular disease (Brian et al., 2014). With aging, the function of vascular endothelial cells diminishes and the elasticity of the blood vessel wall weakens, leading to the alterations in vascular structures and functions (Ungvari et al., 2018). Drugs (e.g., ALT‐711 and MitoQ) can improve endothelial functions and arterial stiffness in healthy middle‐aged and elderly individuals for anti‐aging purpose (Kass et al., 2001; Murray et al., 2022).

Animal models play a crucial role in testing the safety and efficacy of drugs before advancing to humans clinical trials (Tsang et al., 2016). Rodents have the advantage of a short reproductive cycle and are relatively economical. However, rodents have significant physiological differences from humans (José et al., 2012; Meir & Leitersdorf, 2004). For example, in mice (*Mus musculus*), the levels of low‐density lipoprotein and very low‐density lipoprotein that contribute to human atherosclerosis are lower (Meir & Leitersdorf, 2004). Moreover, the majority of atherosclerotic plaques in mice mainly occur at the aortic root, which is different from humans (José et al., 2012). Non‐human primates can develop coronary artery fibro‐fatty atherosclerotic plaques similar to those found in humans when fed a high‐cholesterol diet (Getz & Reardon, 2012). One of the challenges hindering the widespread use of non‐human primate animal models is the high maintenance costs and extended growth cycles (Shim et al., 2016). Three‐dimensional (3D) in vitro models offer advantages, such as reproducibility, high‐throughput capability, and controllability. However, they suffer from inadequate mechanical properties and challenges in mimicking the progression of disease states (Mallone et al., 2021; Pollet & Den Toonder, 2020). For example, cell‐laden hydrogels based on collagen and fibronectin can recapitulate the layered structure of human blood vessel walls (Chen et al., 2006). They still faced challenges such as poor reproducibility and inadequate mechanical properties. The vascular chip system can replicate vascular structures, providing a spatial environment akin to diseased conditions with controllable mechanical stimuli. However, there is little research exploring the methods to induce late‐stage atherosclerotic plaques at present (Mallone et al., 2021). Spherical models of atherosclerosis are difficult to faithfully simulate the layered structure of atherosclerotic blood vessels (Chen et al., 2022).

**FIGURE 1:**
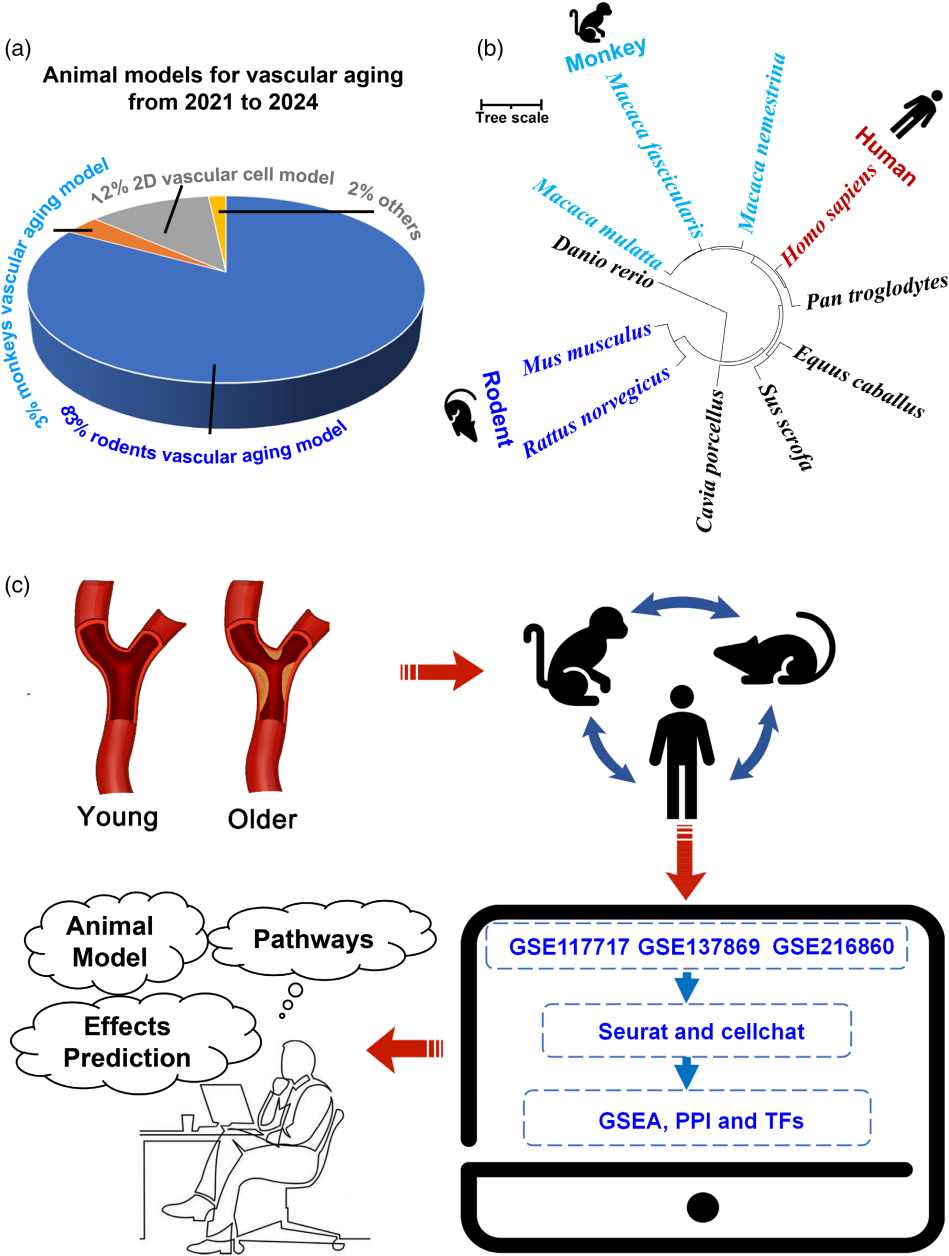
Cross‐species single‐cell transcriptome analysis inspires animal model selections for drug screening. (a) Animal models have been used for vascular aging studies from 2021 to 2024. (b) Species phylogenetic relationship analysis. (c) Schematic diagram of cross‐species single‐cell transcriptome analysis.

Both in vivo and in vitro models have significant limitations in terms of predictability and reliability (Table S1). Among them, rodents were widely used animal models for vascular diseases (Figure 1a). However, the analysis of phylogenetic relationships indicated greater similarity between monkeys and humans, compared with rodents (Figure 1b). Therefore, setting up a reliable algorithm to select a suitable animal model capable of accurately predicting humans cardiovascular dysfunction remains a great challenge (McGonigle & Ruggeri, 2014). In this study, we performed a comparative analysis of the single‐cell transcriptomes of blood vessels in rats (*Rattus norvegicus*), monkeys (*Macaca fascicularis*), and humans (*Homo sapiens*) (Figure 1c). The results showed there were several genes and signaling pathways with consistent/distinct expression trends among rats, monkeys, and humans. Furthermore, an approach called “Cross‐species signaling pathway analysis” has been established and proven to be valuable in selecting appropriate animal models for drug research, confirmed by the consistent trends and the published drug screening results across three species. All the results indicated that our analytical approach held the potential to boost the selections of the animal models for the simulation of humans physiologically and pathologically.

## 2 MATERIALS AND METHODS

### 2.1 Animals experiments and bulk RNA‐seq sequencing

Male C57BL/6 mice were purchased from Cavens‐Biomart (Changzhou, China). Additionally, 6‐week‐old male C57BL/6 mice were used as young mice; while 6‐month‐old male C57BL/6 mice were used as aged mice. All mice were housed under controlled conditions (24°C ± 1°C, 12‐h light/dark cycles, 55% humidity, and specific pathogen–free) and provided with free access to food and water. Before the experiment, the mice in the animal facility were given a 1‐week adaptation period. Animal experiments were performed according to protocols approved by the Ethics Committee of Shanghai University (YS2023‐038), which followed the ethical policies and guidelines on animal testing and research established by the China Animal Protection Committee. The mice were euthanized by cervical dislocation under anesthesia with xylazine (10 mg/kg). The adipose tissue around the aorta was dissected under a stereoscopic microscope. Blood vessels, approximately 2 cm in length, were collected from each mouse, snap frozen in liquid nitrogen, and stored at −80°C. Subsequently, the samples were sent to Shanghai Jingneng (Shanghai, China) for RNA sequencing. High‐throughput sequencing (HTS) was then performed at a minimum depth of ~50 million sequences per mRNA library. A single‐ended 50‐cycle sequencing strategy was used.

### 2.2 Animal models and species phylogenetic relationship analysis

The animal model statistics were obtained through analyzing the types of animal models used in the top 1000 cited articles related to “vascular aging” from 2021 to 2024 in the Web of Science citation database. OrthoVenn3 has been developed as a powerful, web‐based tool that enables users to efficiently infer phylogenetic relationships across a range of species (Sun et al., 2023). The phylogenetic relationships in this article were constructed using OrthoVenn3.

### 2.3 Collection of transcriptome data

The dataset in GEO database (Edgar et al., 2002) offered a number of valuable sources for mining the data of drug study and disease modeling. To verify the reliability of GEO database for the consistency among different RNA‐seq datasets of the same disease in identical animal models, the bulk‐seq datasets with GSE164585 and GSE145972 datasets were compared, showing the results aligned with clinical observations (Table S2) (Björkegren & Lusis, 2022; Libby, 2021). Downstream analysis of the scRNA‐seq datasets was performed using the “Seurat V4” R package developed by the New York Genome Center (Hao et al., 2021).

### 2.4 Gene set enrichment and protein–protein interaction analysis

In drug design, key proteins within a specific signaling pathway are often targeted as they play a crucial role in causing physiological or biochemical effects in cells (Gasek et al., 2021). In order to set up an evaluation criterion, it was investigated whether there is a consistent trend of a specific signaling pathway during vascular aging in rats, monkeys, and humans. Drugs targeting this consistent pathway had a good anti‐aging effect in clinical practice. Conversely, if a target pathway of the drug was not fully activated or inhibited in rats, monkeys, and humans, then selecting an appropriate animal model that closely resembled humans may lead to a better clinically relevant outcome of drug screening.

Gene set enrichment analysis (GSEA) was performed with the use of the Broad Institute algorithm GSEA 4.3.2 on a pre‐ranked list of genes defined from the differential expression analysis results (Subramanian et al., 2005). The enrichment score (ES) can reflect the position of a gene set (at the top or bottom of the overall ranking list). Among them, Normalized enrichment score (NES) is a normalized enrichment score of ES for each gene set. In addition, the GSEA enrichment method can also use positive (+) and negative (−) scores to estimate the significance level of gene sets. Therefore, the + and − values of NES have been used to estimate the activation or inhibition status of the pathway, respectively. The “clusterProfiler” R package was used to visualize the enrichment results (Yu et al., 2012).

The STRING database is an online software where protein–protein interaction (PPI) networks functional enrichment analysis can be performed (Szklarczyk et al., 2015). The DEGs were uploaded to the STRING database (http://string‐db.org/) to construct the PPI network. Cytoscape software (version 3.8.0) was used to visualize the interaction network. In addition, betweenness centrality (BC) algorithm in Cytoscape plug‐in cyto‐NCA was used to identify key genes.

### 2.5 Normalization of single‐cell RNA‐seq (scRNA‐seq) data

Single‐cell RNA‐seq data were massive. Therefore, the dimensionality of the dataset was first reduced. The first step in dimensionality reduction was to select highly variable genes (HVG) as feature selection (Blondel et al., 2008). After feature selection, the dimension of the single‐cell expression matrix was generally further reduced through principal component analysis (PCA) (Pearson, 2010). Clustering was the process of grouping cells based on the similarity of their gene expression profiles. The standardization method used for clustering the single‐cell dataset was the community detection algorithm (Weber & Robinson, 2016).

### 2.6 Clustering and differential expression analysis of scRNA‐seq data

The UMAP algorithm is a visualization dimension reduction technique that requires (approximate) k‐nearest neighbor computation (Pearson, 2010). The resolution of 0.1 was chosen as the optimal resolution for rats, monkeys, and humans to maximally separate the aging cell population from the young cell population (Figure S1). The FindAllMarkers function in Seurat (v4) was used with default parameters to identify marker genes for each cluster. The major cell types were classified by the PanglaoDB website (https://panglaodb.se/) and by the expression of known cell type marker genes (Figures S2 and S3). Differentially expressed genes with log_2_ [fold change] ≥ 0.1 and *p* value ≤ 0.05 were identified using the “FindMarkers” function, employing the “Wilcox” test with Bonferroni correction and log‐transformed fold changes.

### 2.7 Cell–cell communication analysis

CellChat (v1.6.1) is an open‐source R package designed for inferring cell–cell interactions. This R package is based on CellChatDB, a very comprehensive database of ligand–receptor interaction information built manually by Jin et al. (2021). Intercellular communication probability calculation is the main content of CellChat. Equation (1) was used to calculate the ensemble average expression of signal transduction genes in a given group of cells to account for noise effects.

(1) $$\mathrm{EM}=\frac{1}{2}Q_{2}+\frac{1}{4}\left(Q_{1}+Q_{3}\right),$$

where *Q*_1_, *Q*_2_, and *Q*_3_ were the first, second, and third quartiles of the expression levels of signal genes in a cell group.

CellChat projects gene expression profiles onto protein–protein networks based on a random walk propagation method (Paushter et al., 2018). Here, the way in which the communication probability *p*_*i*,*j*_ models *K* from cell group *i* to a particular ligand–receptor of *j* was calculated from Equations (2) and (3). In general, all ligand–receptor pairs were represented by a three‐position array *p* (*K* × *K* × *N*), where *K* was the number of cell populations and *N* was the number of ligand–receptor pairs or signaling pathways. The communication probability of a signaling pathway was calculated by calculating the sum of receptor–ligand pairs associated with it.

(2) $$p_{i,j}^{k}=\frac{L_{i}R_{j}}{K_{h}+L_{i}R_{j}}\times \left(1+\frac{AG_{i}}{K_{h}+AG_{i}}\right)\cdot \left(1+\frac{{\mathrm{AG}}_{j}}{K_{h}+AG_{j}}\right)\times \frac{K_{h}}{K_{h}+AN_{i}}\cdot \frac{K_{h}}{K_{h}+AN_{j}}\times \frac{n_{i}n_{j}}{n^{2}}$$

(3) $$L_{i}=\sqrt[{m1}]{L_{i,1}\cdots L_{i,m1}},R_{j}=\sqrt[{m^{2}}]{R_{j,1}\cdots R_{j,m2}}\cdot \frac{1+RA_{j}}{1+RI_{j}}$$

Here *L*_*i*_ and *R*_*j*_ represented the expression levels of ligand *L* and receptor *R* in cell groups *i* and *j*, respectively. RA and AG represented the mean expression of costimulatory receptors and soluble agonists, respectively. In addition, the proportion of each cell group was also included in the probability calculation, where *n*_*i*_ and *n*_*j*_ are the cells in cell group *i* and cell group *j*, respectively.

### 2.8 Transcription factors analysis

The primary analysis workflow of pySCENIC involves establishing transcription factor–target gene regulatory relationships, recognizing transcription factor binding motifs, and scoring transcription factor activity (Van de Sande et al., 2020). Hg38 was used as the reference genome database for monkeys and humans transcription factors. Mm10 was used as the rat reference genome database for transcription factors. Gradient boosting machine (GBM) algorithm was used to establish the regulatory relationship between transcription factors and target genes. GBM is a machine learning algorithm (Friedman, 2001). The basic idea of GBM algorithm is described below:

(4) $$F_{m}\left(x\right)=F_{m-1}\left(x\right)+\underset{h\in H}{\text{argmin}}\text{Loss}\left(y_{i},F_{m-1}\left(x_{i}\right)+h\left(x_{i}\right)\right).$$

*F*_*m*−1_ (*x*) represented the training sample *x*_*i*_, *y*_*i*_ (*i* = 1, …, *n*) cumulative model obtained at round *m*−1. The second term in the table indicated that a weak learner *h*(*x*) was found in the function space *h*, so that the loss of the *m* round cumulative model was minimized after adding this weak learner.

GBM built a powerful predictive model by combining multiple weak learners. The training process used gradient‐boosting technique to gradually improve the prediction ability of the model. In each round of iteration, the new weak learner was trained to correct the errors of the previous round model to reduce the residual of the model on the data.

### 2.9 Statistical analysis

All statistical analyses were performed using R software (v.4.3.0) (R Core Team, 2020). The student's *t*‐test or Wilcoxon test was used to analyze differences between the two groups. A significance level of *p* < 0.05 was considered statistically significant.

## 3 RESULTS

### 3.1 Cellular composition and interaction analysis of the vascular cells in rats, monkeys, and humans reveals similarities and differences among these three animal models

**FIGURE 2:**
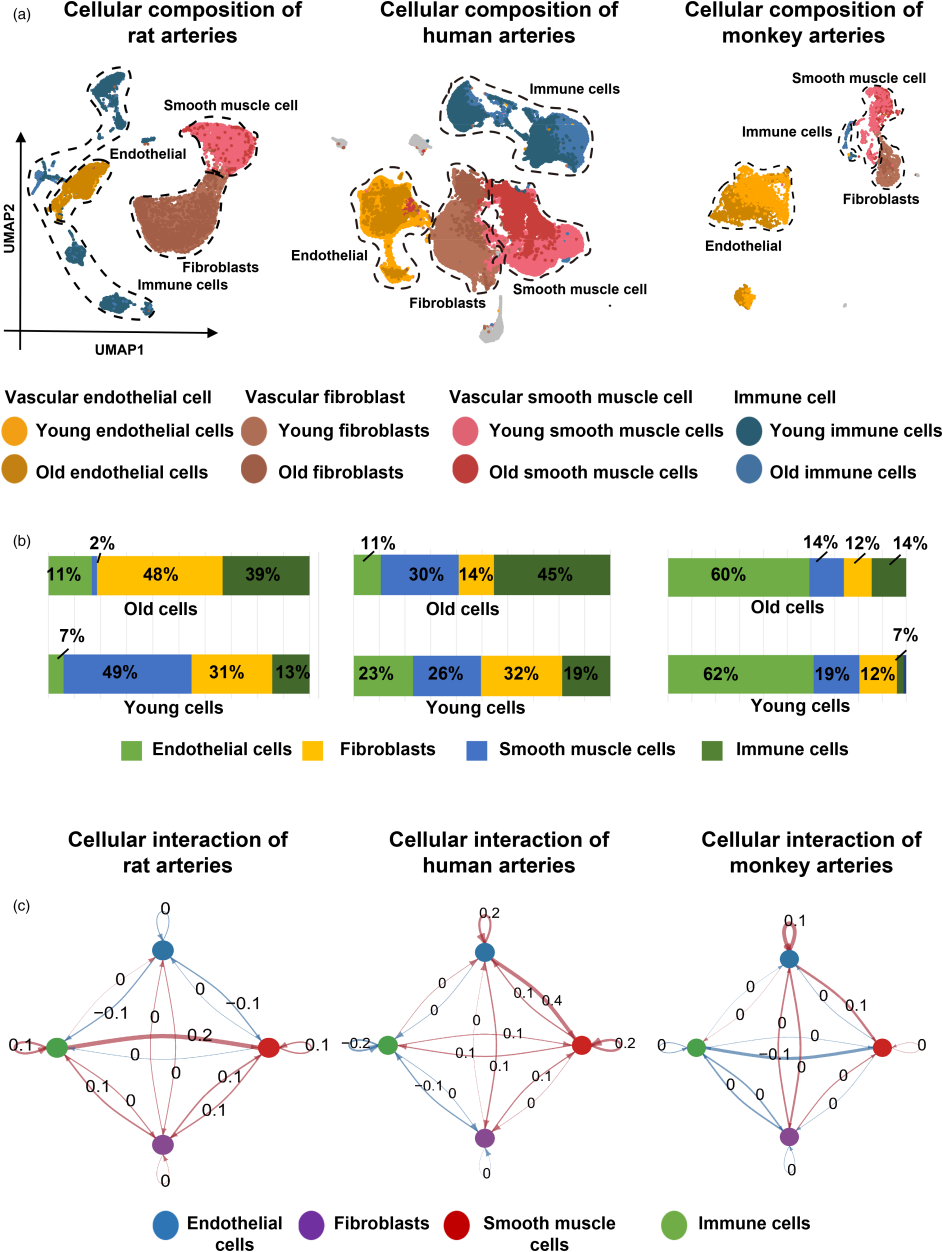
Cellular composition and interaction analysis of the vascular cells in rats, monkeys, and humans. (a) Uniform manifold approximation and projection (UMAP) plot shows the distribution of vascular cells in rats, monkeys, and humans. (b) Bar plots display the percentage of young and old vascular cells in rats, monkeys, and humans. (c) Circle plot highlights the differences in interaction intensity between old and young cells in rats, monkeys, and humans. The red lines represent increased interaction strength in old cells; while the blue lines represent decreased interaction strength in old cells.

Single‐cell transcriptome analysis identified four major vascular cell populations: vascular fibroblasts (FBs), vascular smooth muscle cells (SMCs), vascular endothelial cells (ECs), and immune cells (IMMs) (Figure 2a). Among these, vascular FBs accounted for the largest proportion in rats and humans, at 42% and 26% respectively (Figure S3B). Vascular ECs constituted the smallest proportion in both rats and humans; while being the largest proportion in monkeys (Figure S3B). Additionally, the increased population of IMMs in aged individuals of rats, monkeys, and humans highlights the importance of IMMs in vascular aging, consistent with multiple previous reports (Figure 2b) (El Assar et al., 2013; Koutsaliaris et al., 2022; Liberale et al., 2022).

To investigate the primary cell populations involved in vascular aging, we proceeded with CellChat analysis (Figure 2c). The results indicated that both the total number and strength of cell interactions increased in aging cells of rats, monkeys, and humans (Figure S4). By comparing the output and input signals of vascular FBs, vascular SMCs, vascular ECs and IMMs in rats, monkeys, and humans (Figure S4), the results showed that vascular FBs had the highest sum of input and output in aging individuals of humans and rats; while vascular ECs had the highest sum of input and output in aging monkeys.

### 3.2 The genes encoding collagen proteins were upregulated during vascular aging in rats, monkeys, and humans

**FIGURE 3:**
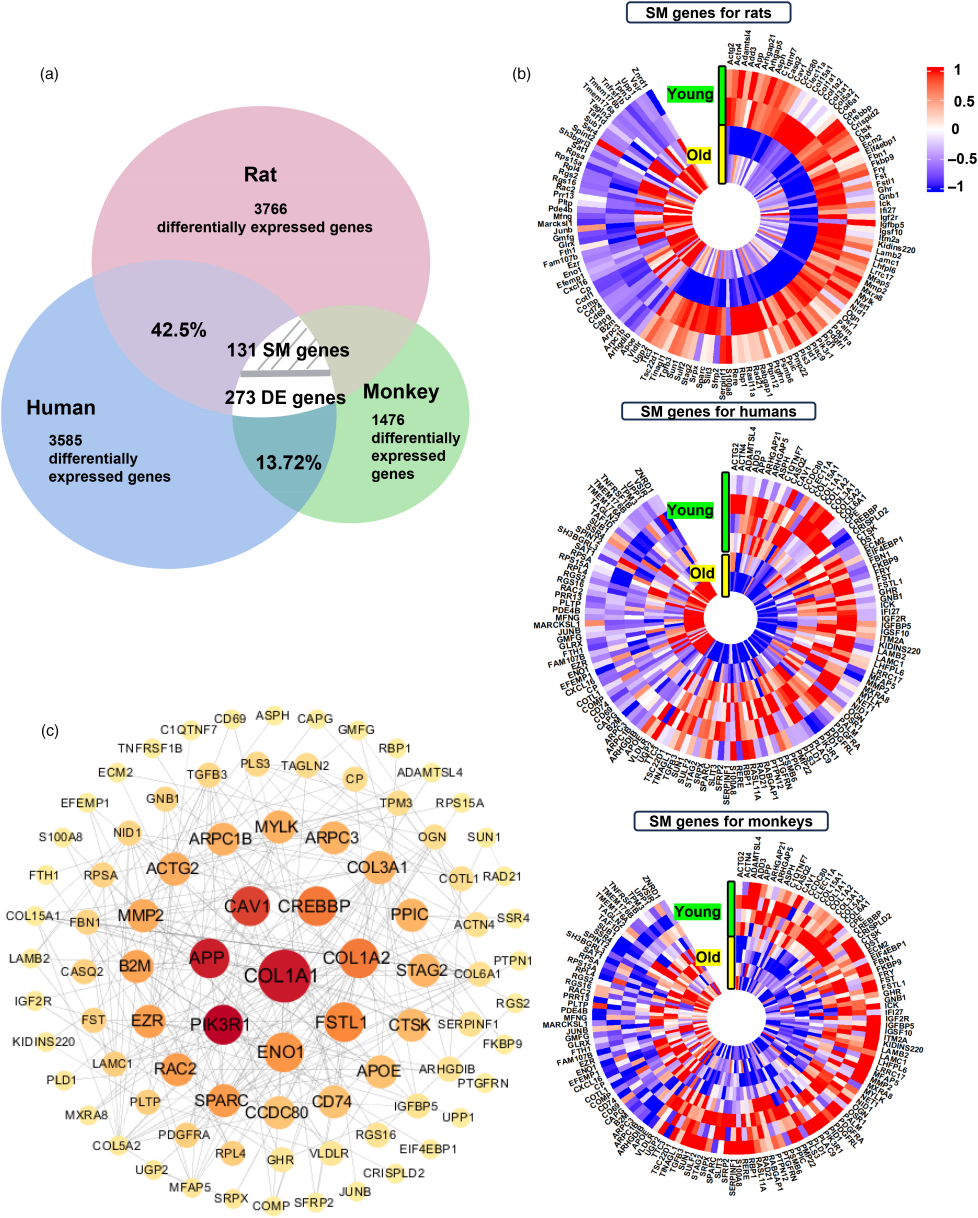
The genes encoding collagen proteins are upregulated during vascular aging processes in multiple species. (a) The Venn plots show the overlapping number of differentially expressed genes in rats, monkeys, and humans with fold change >0.1 or <−0.1, *p* value < 0.05. Among the overlapping differentially expressed genes, the genes with the same relative expression trend between the young and old groups were defined as “same genes (SM genes)”; while the genes with the different expression trends were defined as “different genes (DE genes).” (b) Heatmap presents the 131 SM genes in rats, monkeys, and humans. (c) Protein–protein interaction networks (PPI) based on 131 SM genes in rats, monkeys, and humans.

Rats and monkeys had 3766 (42.5% of human differentially expressed genes) and 1476 (13.72% of human differentially expressed genes) differentially expressed genes associated with vascular aging, respectively (Figure 3a). Among the overlapping differentially expressed genes, the genes with the same relative expression trend between the young and old groups were defined as “same genes (SM genes)”; while the genes with the different expression trends were defined as “different genes (DE genes).” 131 SM genes showed the same relative expression trend among all overlapping differentially expressed genes in rats, monkeys, and humans (Figure 3b). We constructed a PPI network of these 131 SM genes that overlapped in rats, monkeys, and humans. The results showed that COL1A1 was one of the most significant central proteins in the PPI network, indicating its significant role during vascular aging (Figure 3c).

The COL1A1 gene encodes collagen and glycosylated cross‐linking of collagen can accumulate in senescent vascular cells, leading to vascular sclerosis (Deguchi et al., 2005; Vafaie et al., 2014). Our results indicated the importance of COL1A1 during vascular aging and suggested that collagen has the potential to be a drug target for delaying vascular aging, with promising clinical efficacy.

### 3.3 Differences in signaling pathways during vascular senescence in rats, monkeys, and humans reveal the importance of cross‐species transcriptome analysis in guiding animal model selection

**FIGURE 4:**
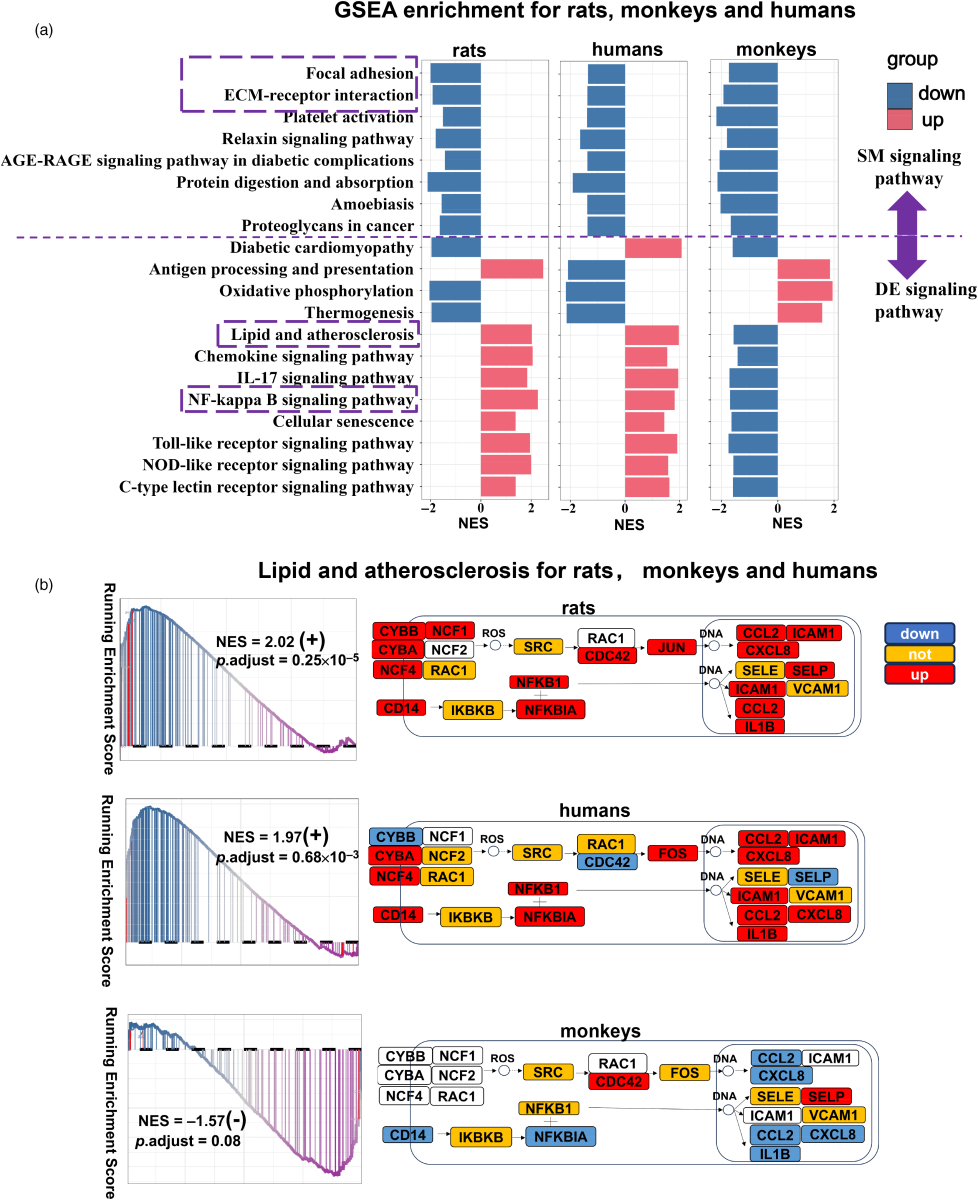
Differences in signaling pathways during vascular aging in rats, monkeys, and humans. (a) Bar plots display overlapping pathways among rats, monkeys, and humans. The pathways above the dashed gray line represent SM signaling pathways (a signal pathway with the same NES symbol); while the pathways below the dashed gray line represent DE signaling pathways (a signal pathway with a different NES symbol). (b) The pathway diagram shows the expression of the “Lipid and atherosclerosis” (KEGG:05417) signaling pathway in blood vessel cells of rats, monkeys, and humans. Red boxes indicate the upregulation in old cells. Blue boxes indicate downregulation in old cells. Yellow boxes indicate no change.

The results of GSEA enrichment in rats, monkeys, and humans revealed 83 overlapping gene sets (79% in total gene sets) between rats and humans; while 38 overlapping gene sets (36% in total gene sets) were found between monkeys and humans (Figure S7B). The pathways with consistent NES symbols were defined as “SM signaling pathways”; while those with inconsistent NES symbols were labeled as “DE signaling pathways.” Notably, we observed that both the pathways of “ECM‐receptor interaction” (KEGG:04512) and “Focal adhesion” (KEGG:04510) were consistently downregulated in the aged aorta of rats, monkeys, and humans (Figure 4a and Table S5). Additionally, “IL‐17 signaling pathway” (KEGG:04657), “Lipid and atherosclerosis” (KEGG:05417), “Cellular senescence” (KEGG:04218) and “NF‐kappa B signaling pathway” (KEGG:04064) were activated (old/young NES = +) in senescent vascular cells in both rats and humans; while they were inhibited (old/young NES = −) in monkeys (Figure 4a and Table S5).

The COL1A1 gene encoding collagen is involved in the “ECM‐receptor interaction” and “Focal adhesion” signaling pathway. Glycosylated cross‐linked collagen is deposited in aging blood vessels (Susic et al., 2004). ALT‐711 and resveratrol are two major drugs that delay vascular aging by interfering with the degree of collagen cross‐linking (Csiszar et al., 2012; Rungratanawanich et al., 2021; Vaitkevicius et al., 2001; Vasan et al., 1996). From the literature, ALT‐711 has demonstrated consistent cardiovascular benefits in rats, monkeys, and humans. Another drug, resveratrol, has similar effects (Table 1). Therefore, the good clinical results of these two drugs may be due to the fact that the targeted signaling pathways were consistent in animal models and humans.

**TABLE 1:** Comparative efficacy of anti‐vascular aging drugs in rats, monkeys, and humans.

Category	Targeted pathways	Model	Drug	Result	References
SM	ECM‐receptor interaction	Rats	ALT‐711	Reversing the increase in arterial stiffness	Wolffenbuttel et al. (1998)
	ECM‐receptor interaction	Monkeys	ALT‐711	Improving arterial and ventricular function	Vaitkevicius et al. (2001)
	ECM‐receptor interaction	Humans	ALT‐711	Cardiovascular benefits	Kass et al. (2001)
	Focal adhesion	Rats	Resveratrol	Delaying vascular aging	da Luz et al. (2012)
	Focal adhesion	Monkeys	Resveratrol	Delaying vascular aging	Csiszar et al. (2012)
	Focal adhesion	Humans	Resveratrol	Delaying atherosclerosis	Micans (2013)
DE	Lipid and atherosclerosis	Rats	MitoQ	Reversing endothelial dysfunction	Gioscia‐Ryan et al. (2018), Gioscia‐Ryan et al. (2014)
	Lipid and atherosclerosis	Monkeys	MitoQ	Only be effective in 23–25‐year‐old animals	Klots et al. (2022)
	Lipid and atherosclerosis	Humans	MitoQ	Delaying atherosclerosis	Murray et al. (2022), Rossman et al. (2018)
	NF‐kappa B signaling pathway	Rats	Salicylic acid	High embryotoxicity	Wilson et al. (1977)
	NF‐kappa B signaling pathway	Monkeys	Salicylic acid	Low embryotoxicity	Wilson et al. (1977)
	NF‐kappa B signaling pathway	Humans	Salicylic acid	Improving vascular endothelial	Labib et al. (2018)

Abbreviations: DE, signaling pathways with opposite relative expression trends; SM, signaling pathways with consistent relative expression trends.

Aging vessels are also associated with decreased nitric oxide (NO) availability, endothelial dysfunction, oxidative stress, and increased inflammation. MitoQ and salicylic acid can delay the aging process by reversing the phosphorylation of nuclear factor kappa B, reducing the production of ROS by mitochondria, and eliminating inflammation (Ungvari et al., 2018). Here, we noticed that there was a consistent expression trend in rats and humans (but not monkeys) for the pathway of “Lipid and atherosclerosis” (KEGG:05417) and the “NF‐kappa B signaling pathway” (KEGG:04064) (Figure S8A). The literature indicated that MitoQ was only effective in monkeys aged 23–25 years. It was not useful or even harmful in old animals (Klots et al., 2022). Moreover, there have been no reported adverse developmental effects in clinical trials up to the maximum recommended therapeutic dose for salicylic acid, which was similar to the results in the rat model, but not in the monkey model (Table 1).

In conclusion, when selecting the animal models for preclinical studies, it was necessary to analyze the consistency between the animal models (e.g., rodents or monkeys) and humans. Specifically, we needed to carefully select animal models when we were choosing “DE signaling pathways” in rats or monkeys; otherwise, it might have led to the failure of drug clinical trials. On the other hand, it is practical to use either rats or monkeys as the animal model for the signaling pathway with consistent NES symbols to humans.

### 3.4 Prediction of potential novel targets for anti‐vascular aging drugs with cross‐species signaling pathways analysis

The analysis above suggested that rats and humans were more similar in the process of vascular aging compared with monkeys. Based on this, we further compared the similarity of transcription factors (TFs) regulating vascular aging between rats and humans. The results showed that the transcription factor AP‐1 family exhibited significant differences during the process of vascular aging in humans. Among them, JUNB, JUN, ATF3, and FOS were all highly expressed in senescent cells, which were also upregulated in rat senescent transcriptomes. In addition, FOS and JUN were present in the “lipid and atherosclerosis” (KEGG:05417) (SM pathway in rats and humans) (Figure 4b). The study showed that JUN, ATF3, and FOS could regulate cellular senescence by establishing accessible chromatin, which echoed the results we had discovered above (Table 2) (Vierbuchen et al., 2017).

**TABLE 2:** Comparison of experimental results of AP‐1 family members in regulating cellular senescence in rodents and humans.

Key factors	Results	Species	Model	Conclusion	References
ATF3	Reconstructing accessible chromatin profiles	Human	HUVEC	Successful	Zhang et al. (2020)
c‐JUN	Regulation of oncogene‐induced senescence programs	Human	FBs	Successful	Martínez‐Zamudio et al. (2020)
FOS/JUN	Core enhancers for FBs	Rodents	FBs	Successful	Vierbuchen et al. (2017)
JUN	Regulation of HAEC transcription by binding enhancers	Human	HAEC	Successful	Hogan et al. (2017)
JUNB/ATF3	Functions to promote angiogenesis	Rodents	ECs	Successful	Zhong (2023)

Abbreviations: FBs, fibroblasts; HUVEC, human umbilical vein endothelial cells; TFs, transcription factors.

Therefore, transcription factor AP‐1 family, especially FOS, JUN, ATF3, and JUNB, were the key molecules affecting vascular cell senescence. When evaluating drugs that targeted these TFs, rats might have been a more appropriate animal model than monkeys. We also constructed software for searching the vascular aging pathways in rats, monkeys, and humans. Using this software, we could search for the vascular aging pathways overlapped in rats, monkeys, and humans (Figure S9).

## 4 DISCUSSION

Animal models have been widely used for disease study, drug screening, and preclinical validations since the 1900s (Baptista et al., 2021). However, in clinical drug testing, 90% of the drugs failed due to the loss of drug effects or the occurrence of side effects (Sun et al., 2022). One of the reasons for these problems was the significant differences between animal models used in drug screening and the actual physiological levels in humans (Davis et al., 2020; Emmerich et al., 2021). In June 2022, the Food and Drug Amendment Act of 2022 (H.R.7667) was passed by the United States House of Representatives. On the eve of Christmas that year, the FDA Modernization Act 2.0 was officially passed, adding that “FDA no longer listed animal experiments as a necessary part of drug development.” The passage of this bill once again confirmed the long‐standing issue of model safety and efficacy in animal experiments, which differed greatly from clinical argumentation (Alastair Stewart et al., 2023). Signaling pathways are often used as important targets for drug design (Zheng et al., 2020). In this article, an approach called “Cross‐species signaling pathways analysis” was developed to compare the similarities and differences among different models. It was discovered that the signaling pathways (e.g., ECM‐receptor interaction, focal adhesion) were expressed similarly during vascular aging in rats, monkeys, and humans. Additionally, the signaling pathways (e.g., IL‐17 signaling pathway, lipid, and atherosclerosis, NF‐kappa B signaling pathway) were similarly expressed in rats and humans but oppositely expressed in monkeys (Figure 4). Through the analysis, it was found that using rats and monkeys as screening models when targeting SM signaling pathways (such as ECM‐receptor interaction and Focal adhesion) was feasible. However, careful considerations were necessary for choosing the proper models (e.g., rats but not monkeys) when targeting the DE signaling pathways (such as IL‐17 signaling pathway, lipid and atherosclerosis, and NF‐kappa B signaling pathway).

“Cross‐species signaling pathways analysis” revealed that bioinformatics analysis based on disease characteristics can help to select more appropriate animal models in clinical drug trials. Species phylogenetic relationships analysis showed that monkeys and humans were closer in genome composition compared with rodents. However, interestingly, our analysis indicated that rodents were closer to humans in terms of vascular cell composition and vascular cell interactions during the process of vascular aging. Additionally, the differential expressed genes and signaling pathways shared between rodents and humans were more abundant than those shared with monkeys. Therefore, simply relying on phylogenetic relationships to select the most similar animal model could not fully simulate the true characteristics of the disease in models. The development of 3D in vitro models provides a new perspective for selecting biological models (Mallone et al., 2021; Pollet & Den Toonder, 2020).

**FIGURE 5:**
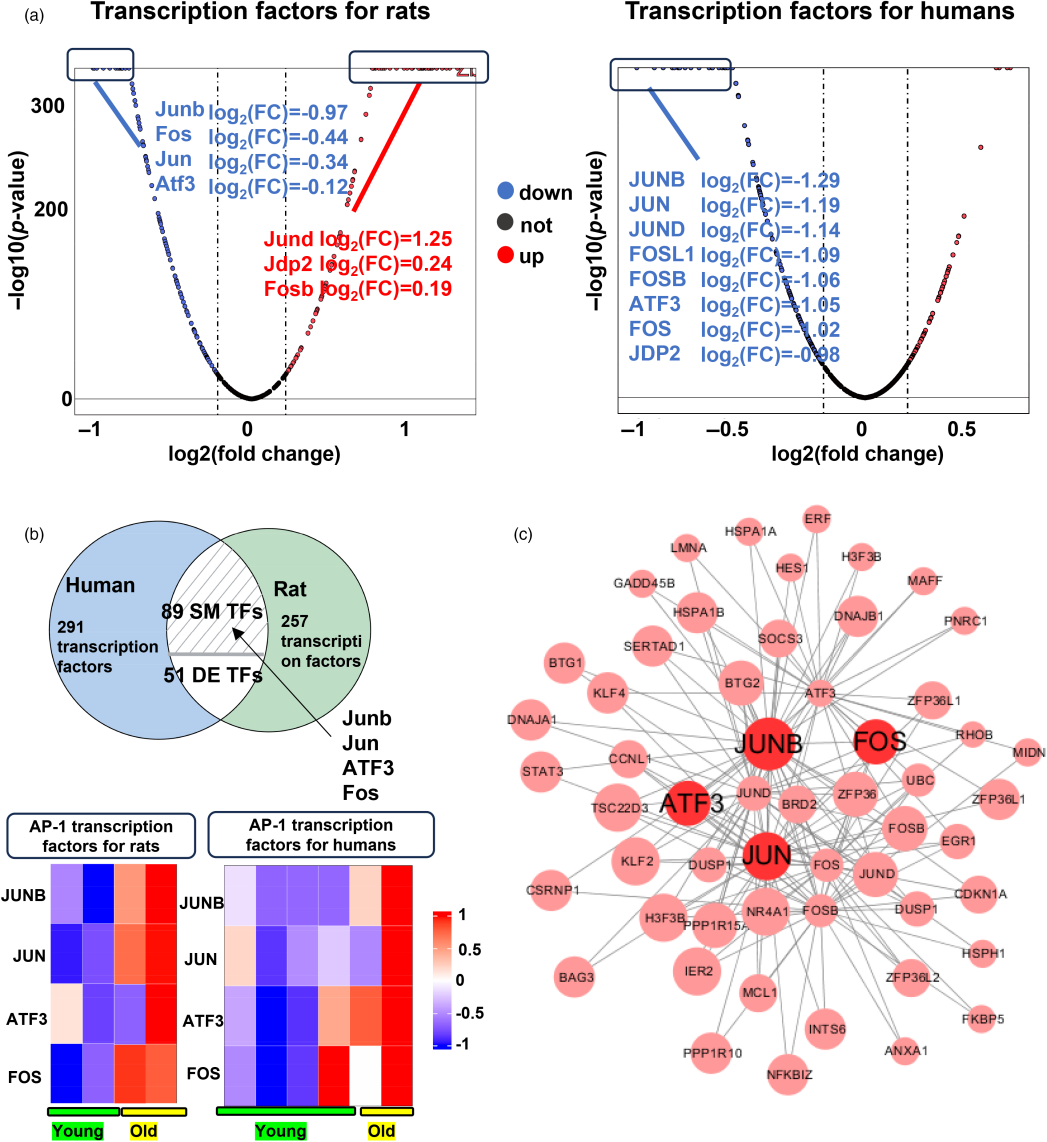
Prediction of potential novel targets for anti‐vascular aging drugs. (a) Volcano plot shows the significantly different transcription factors (TFs) during rats and humans' vascular aging. (b) The Venn plots demonstrate the overlap of TFs in rats and humans with fold change >0.1 or <−0.1, *p* value < 0.05. Heatmap shows FOS, JUN, JUNB, and ATF3 gene expression. (c) Network diagram shows the FOS, JUN, JUNB, and ATF3 regulation of the downstream genes.

Furthermore, “Cross‐species signaling pathways analysis” can also inspire the design of the drugs related to vascular aging. In our study, we found that JUNB, JUN, ATF3, and FOS were significantly upregulated in aged rats and humans vascular cells (Figure 5). Several reports have obtained similar experimental results to support our findings. For instance, Martínez‐Zamudio et al. (2020) reported that AP‐1 can imprint a reversible transcriptional program on senescent cells (Table 2). Chao Zhang et al. (2020) found that the increased openness of chromatin facilitated the binding of the motile transcription factor ATF3, which promoted cell aging. The experimental results from Vierbuchen et al. (2017) showed that FOS/JUN can establish accessible chromatin by synergistically binding with nucleosome enhancers and recruiting the AWI/SNF (BAF) chromatin remodeling complex. Hogan et al. (2017) found that the AP‐1 transcription factor regulated HAEC transcription by binding to enhancers. Additionally, the exposure of HAEC to pro‐inflammatory cytokines led to signal‐specific changes in the enhancer landscape, which were associated with coordinated binding with NF‐κB. Zhong Liu et al. identified a set of key TFs regulating mouse aging‐associated DEGs, including EGR1, JUNB, and ATF3 (Zhong et al., 2023). Therefore, JUNB, JUN, ATF3, and FOS were estimated to be potential targets for anti‐vascular aging therapies. All these facts further validated the value of the approach of “Cross‐species signaling pathways analysis.”

However, this study also had some limitations. Firstly, the work on our proposed “Cross‐species signaling pathways analysis” was a pipeline for analyzing transcriptome data rather than a new algorithm. Secondly, we used a simple Wilcoxon test in the Seurat R package, which was acceptable for understanding cell type‐specific markers but not very comprehensive for differential expression under different conditions (such as old vs. young in this study). In addition, collecting and comparing vascular single‐cell data from more model animals (e.g., pig, rabbit, zebrafish, etc.) might make our cross‐species signaling pathway analysis more meaningful. Finally, we also hope that our cross‐species signaling pathway analysis method can be applied to other disease types, not just the vascular aging diseases in this article.

## 5 CONCLUSIONS

In sum, an approach called “Cross‐species pathway analysis” was developed to select suitable models for drug screening. The effectiveness of this method was validated through the analysis of the pharmacological predictions of four commonly used anti‐vascular aging drugs and their validation through animal/clinical experiments. Finally, the AP‐1 family of TFs, especially JUNB, JUN, ATF3, and FOS were identified as anti‐vascular aging drugs that are consistent with the literature reports. These drug targets for anti‐vascular aging showed promise for further clinical trials. In the future, more algorithms may be needed to understand the differences in transcriptome among different species. Apart from animal models, organ‐on‐chip models also have certain advantages in drug research, including improved accuracy, shortened time, and cost savings.

## CONFLICT OF INTEREST STATEMENT

The authors declare no conflict of interest.

## ANIMAL EXPERIMENT

Male C57BL/6 mice were purchased from Cavens‐Biomart (Changzhou, China). Animal experiments were performed according to protocols approved by the Ethics Committee of Shanghai University (YS2023‐038), which followed the ethical policies and guidelines on animal testing and research established by the China Animal Protection Committee. The mice were euthanized by cervical dislocation under anesthesia with xylazine (10 mg/kg, Meryer, China). Vessel samples were sent to Shanghai Jingneng (Shanghai, China) for RNA sequencing.

## Supporting information


Figure S1.
Figure S2.
Figure S3.
Figure S4.
Figure S5.
Figure S6.
Figure S7.
Figure S8.
Figure S9.

Table S1.
Table S2.
Table S3.
Table S4.
Table S5.

## Data Availability

Data openly available in a public repository (GSE246658). All data supporting the results of this study are available in the article and its supplementary information document, or upon request from the corresponding author. No custom code or mathematical algorithms were used, and the name and version of the software are included in the methods.
