# Supplementary material for: Cross‐species signaling pathways analysis inspire animal model selections for drug screening and target prediction in vascular aging diseases
Source: Evol Appl. 2024 Jun 10;17(6):e13708. doi: 10.1111/eva.13708 (PMC11164676; doi:10.1111/eva.13708)
Supplement: Supplementary file 1 — Figure S1. Figure S2. Figure S3. Figure S4. Figure S5. Figure S6. Figure S7. Figure S8. Figure S9. [file EVA-17-e13708-s001.pdf]

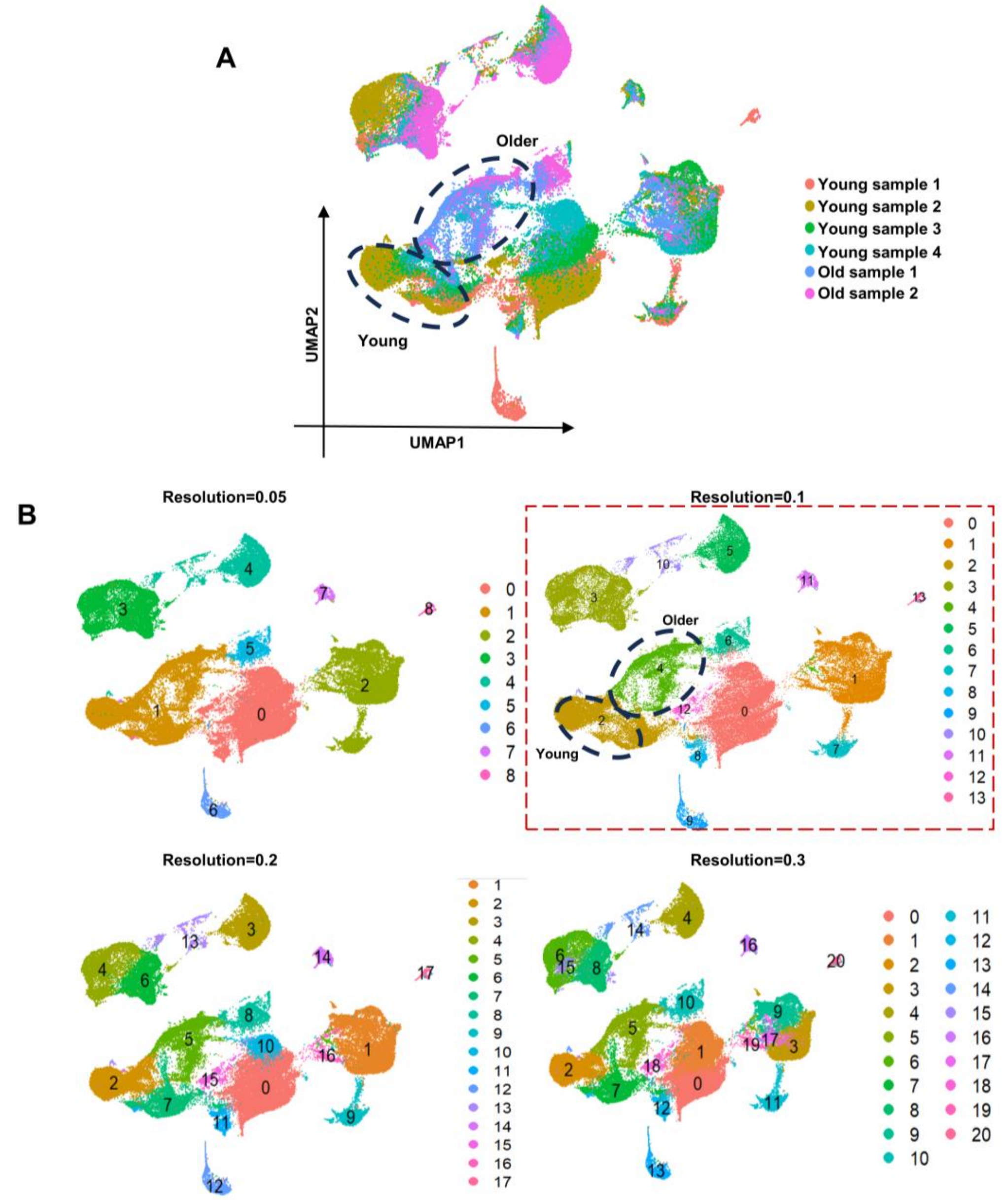

Figure S1. Uniform manifold approximation and projection (UMAP) resolution selection. (A) UMAP plot shows the distribution of cells from different vascular samples in humans. (B) UMAP plot shows the number of clusters under different resolution conditions of 0.05, 0.1, 0.3 and 0.4.

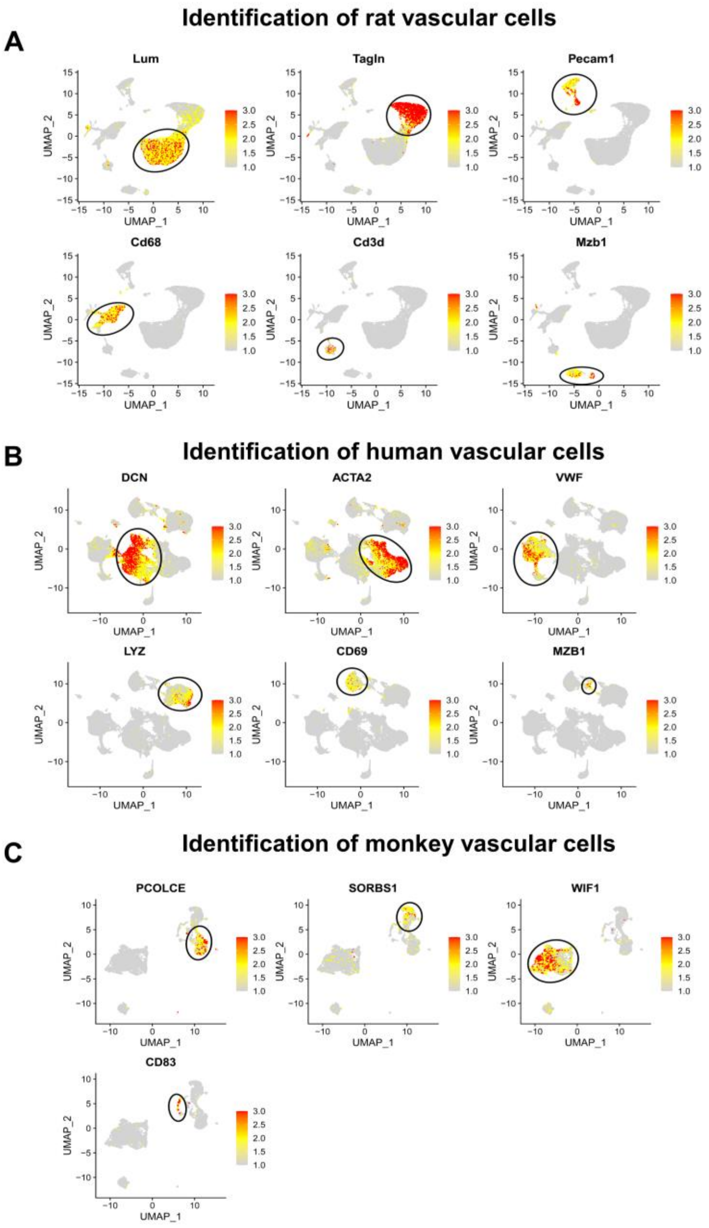

Figure S2. Identification of vascular cells in rats, monkeys, and humans. (A) UMAP plot shows the expression of known identified specific cell markers for rats using the same layout as in Figure 2A (gray, no expression; dark red, relatively higher expression). Lum, Tagln and Pecam1 are the marker genes for rat vascular fibroblasts (FBs), vascular smooth muscle cells (SMCs) and vascular endothelial cells (ECs), respectively. Cd68，Cd3d and Mzb1 are the marker genes for rat vascular immune cells (IMMs). (B) UMAP plot shows the expression of known identified specific cell markers for humans. DCN, ACTA2 and VWF are the marker genes for human vascular FBs, vascular SMCs and vascular ECs, respectively. LYZ, CD69 and MZB1 are the marker genes for human vascular IMMs. (C) UMAP plot shows the expression of known identified specific cell markers for monkeys. PCOLCE, SORBS1 and WIF1 are the marker genes for monkey vascular FBs, vascular SMCs and vascular ECs, respectively. CD83 is the marker gene for monkey vascular IMMs.

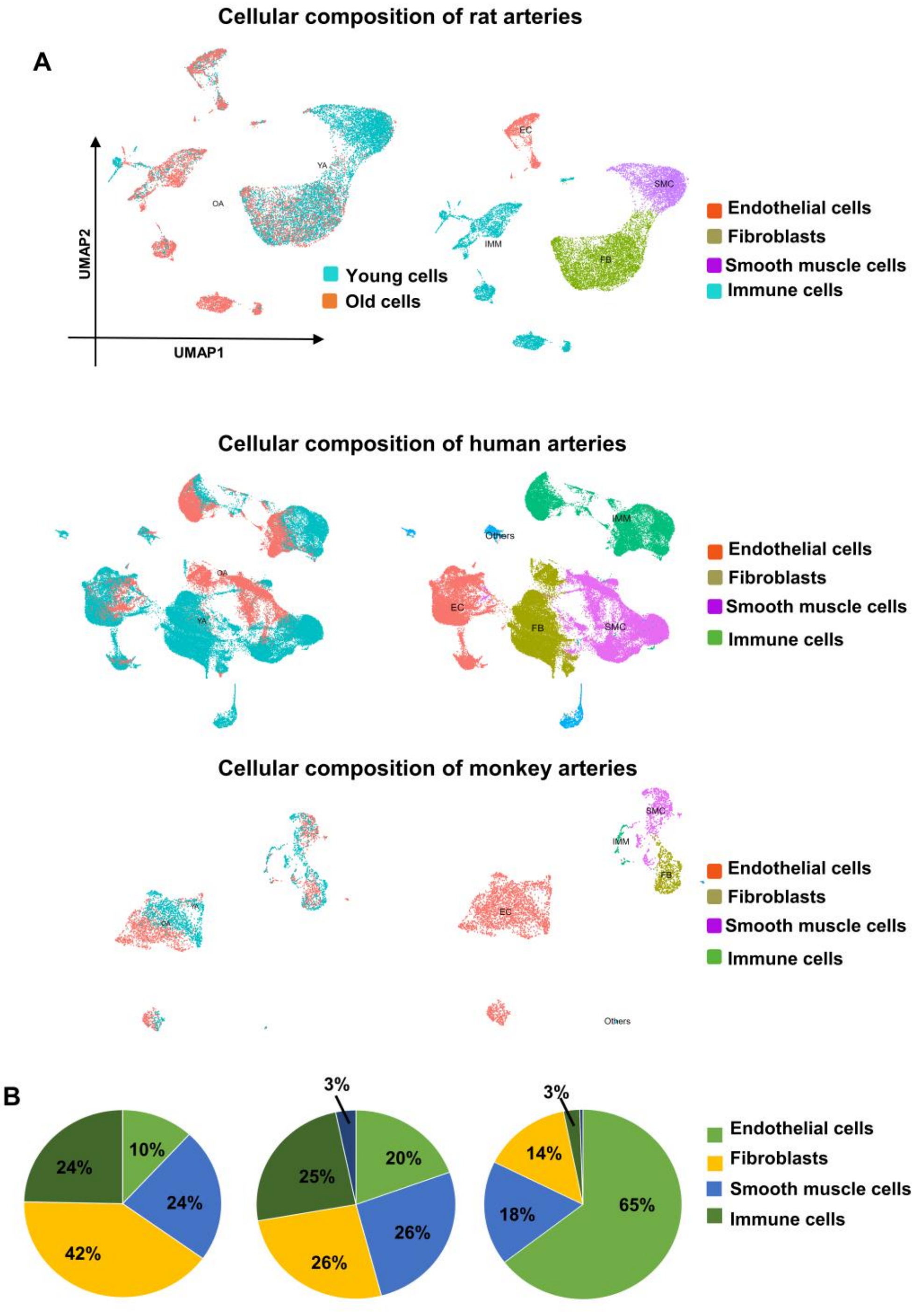

Figure S3. The composition of blood vessel cells, related to Figure 2B. (A) UMAP plots show the composition of blood vessel cells in rats, monkeys, and humans. (B) Pie charts show the proportions of vascular cells types in rats, monkeys, and humans.

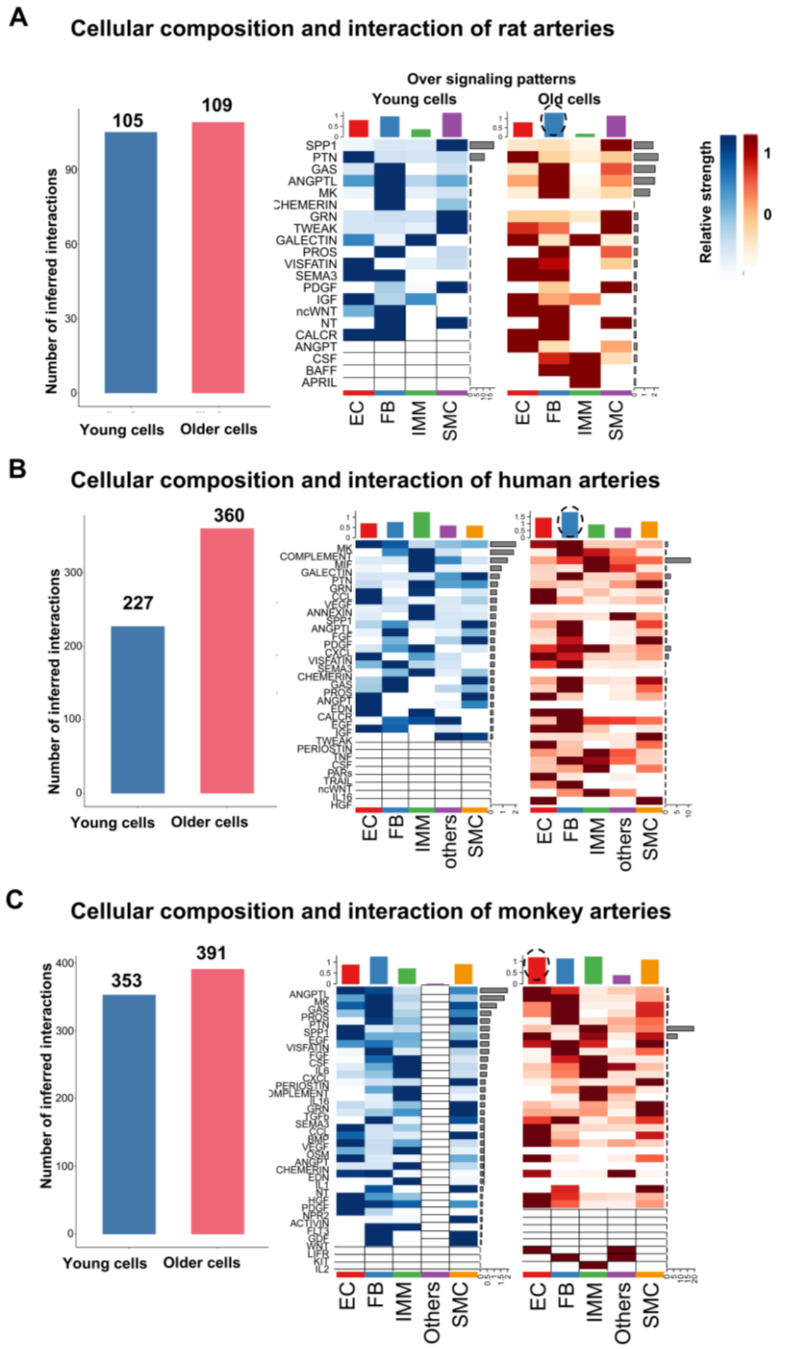

Figure S4. CellChat analysis for vascular single-cell RNA-seq (scRNA-seq) in rats, monkeys and humans, related to Figure 2C. (A) Cellular composition and interactions in rat arteries. Bar plots show the total internal interactions in terms of quantity and intensity in rats. Old cells are represented in red; while young cells are represented in blue. Heatmaps display the overall signaling patterns of transmitting (or incoming) signals from young and older cells in rats. (B) Cellular composition and interactions in human arteries. (C) Cellular composition and interactions in monkey arteries.

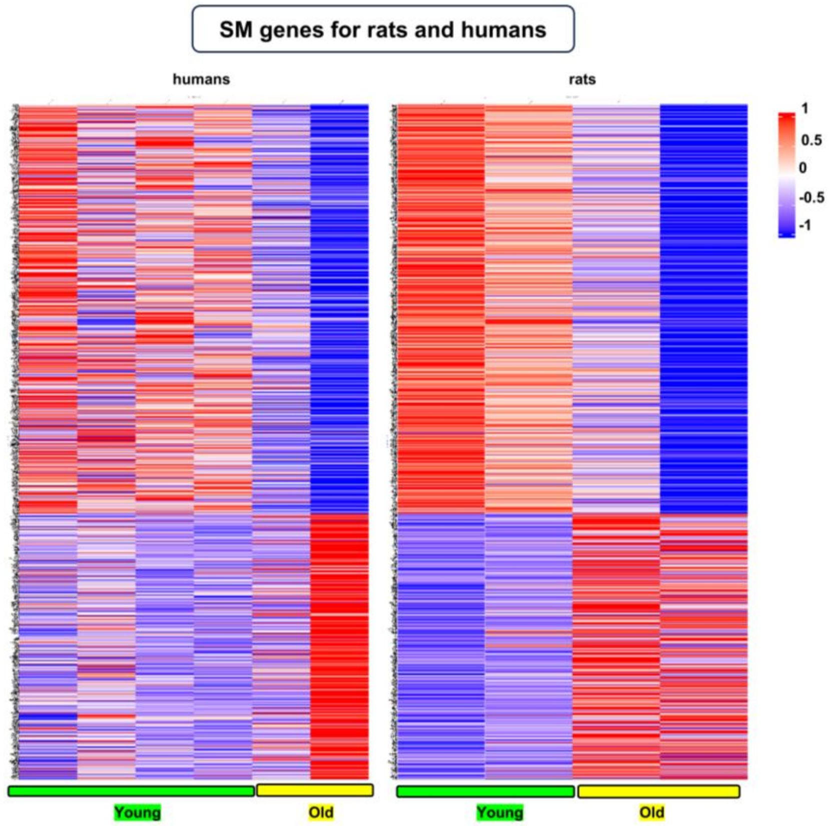

Figure S5. Heatmap presents the genes with the same relative expression trend between the young and old groups (SM genes) in rats and humans. Each cell in the heatmap represents the average expression level of the gene across different samples. The left and right heatmaps show the expression levels of overlapping genes in human and rat, respectively, and the genes are arranged in a consistent order. The bluer the color, the lower the expression level, and the redder the color, the higher the expression level.

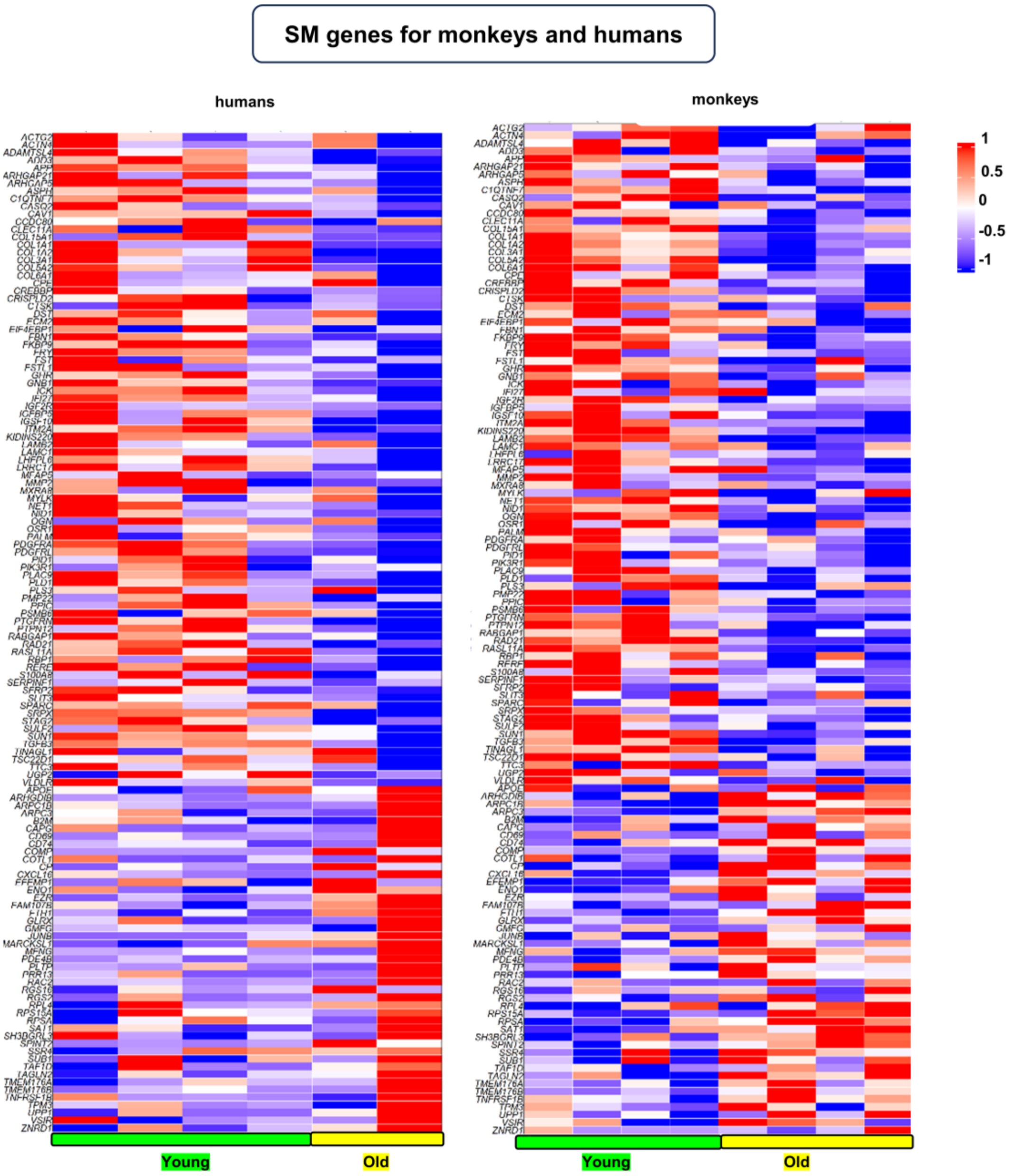

Figure S6. Heatmap presents the SM genes in monkeys and humans. Each cell in the heatmap represents the average expression level of the gene across different samples. The left and right heatmaps show the expression levels of overlapping genes in human and monkey, respectively, and the genes are arranged in a consistent order. The bluer the color, the lower the expression level, and the redder the color, the higher the expression level.

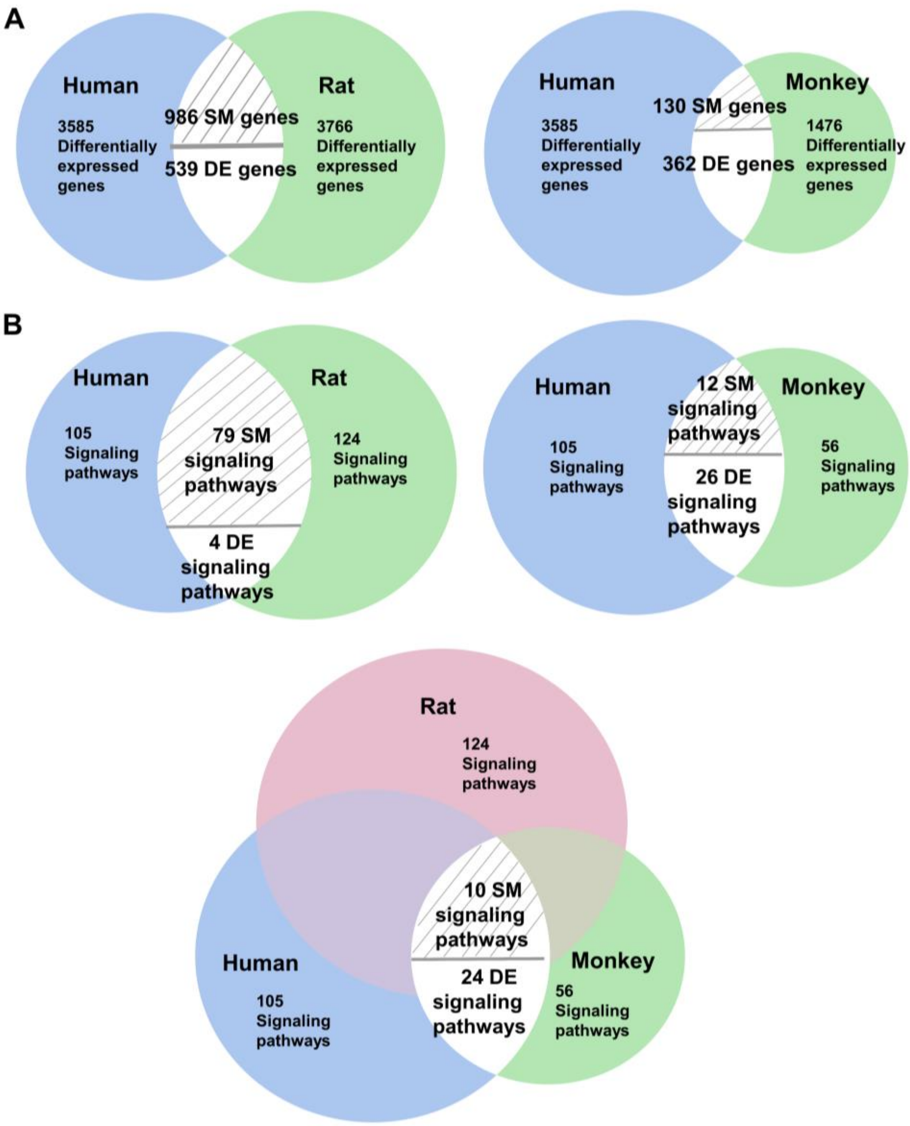

Figure S7. Transcriptome differential expression analysis and GSEA pathway enrichment of young and older vascular cells from rats, monkeys and humans, related to Figure 3 and Figure 4**. (**A) The Venn plots show the overlap number of differential genes in rats, monkeys and humans. The genes with the same relative expression trend between the young and old groups were defined as "same genes (SM genes)"; while genes with the different expression trends were defined as "different genes (DE genes)". (B) The Venn plots show the overlap number of GSEA pathways in rats, monkeys and humans. The pathways with consistent NES symbols were defined as "SM signaling pathways", while those with inconsistent NES symbols were labeled as "DE signaling pathways".

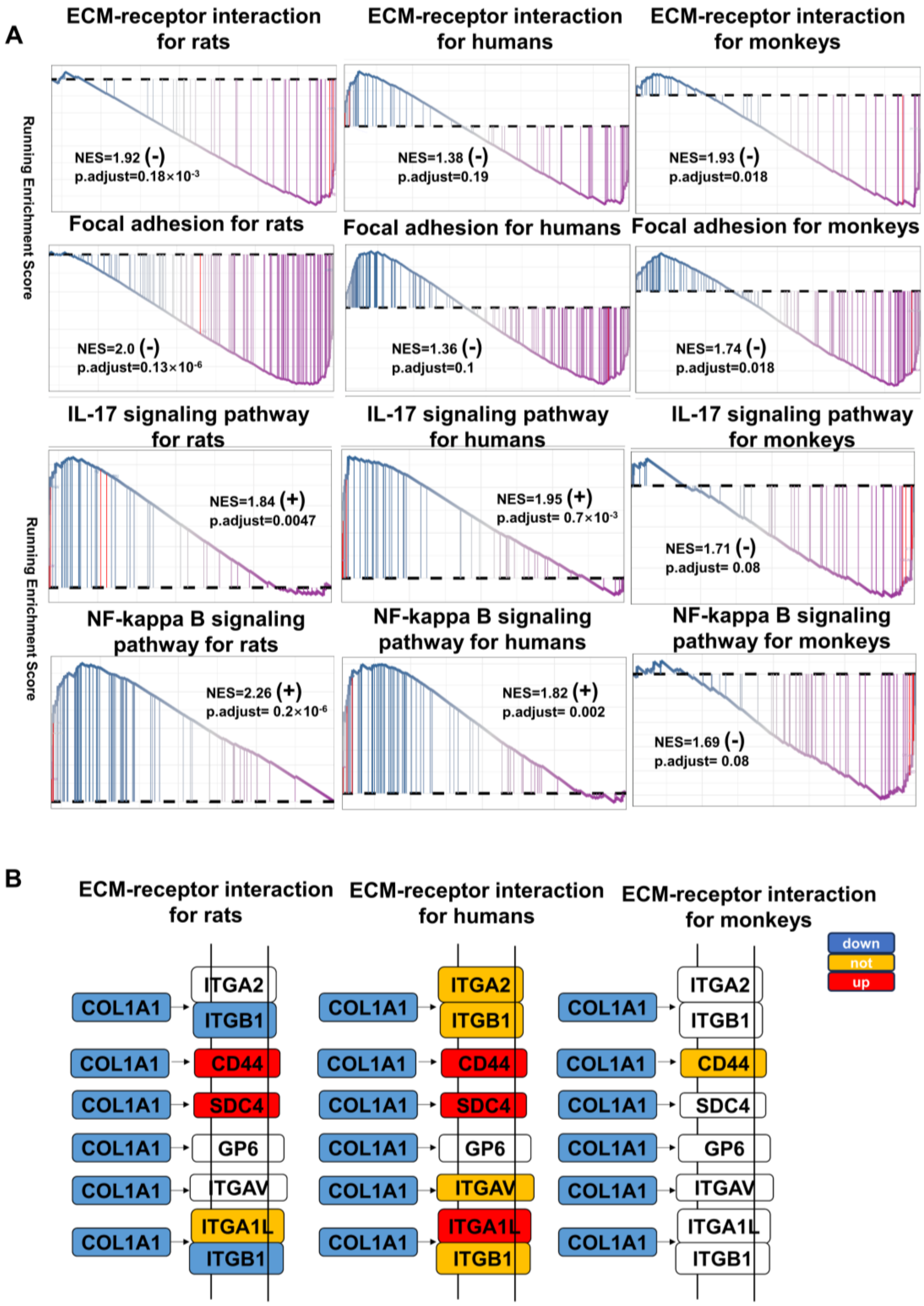

Figure S8. Comparison of GSEA enrichment results of rats, monkeys and humans. (A) The enrichment curve shows the expression of ECM-receptor interaction (KEGG:05412), Focal adhesion pathways (KEGG:04510), IL-17 signaling pathway (KEGG:04657) and NF-kappa B signaling pathway (KEGG:04064) in the vascular transcriptome of rats, monkeys and humans. (B) The pathway diagram shows the expression of the ECM-receptor interaction (KEGG:04512) in rats, monkeys and humans. Red boxes indicate upregulation in old cells, blue boxes indicate downregulation in old cells, and yellow boxes indicate no change.

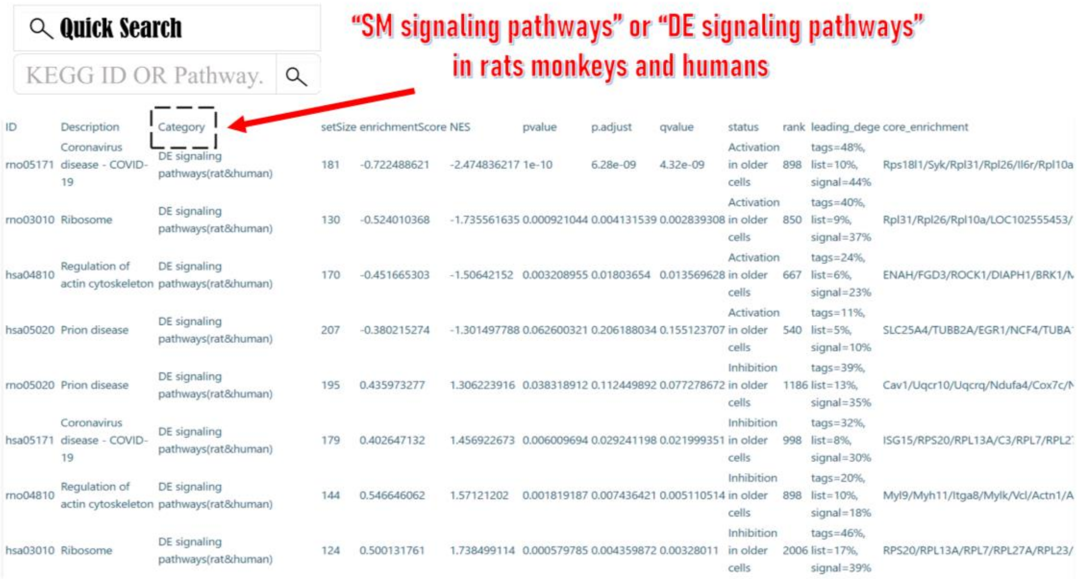

ID	Description	Category	setSize	enrichmentScore	NES	pvalue	p.adjust	qvalue	status	rank	leading_dege	core_enrichment
rno05171	Coronavirus disease - COVID-19	DE signaling pathways(rat&human)	181	-0.722488621	-2.474836217	1e-10	6.28e-09	4.32e-09	Activation in older cells	898	tags=48%, list=10%, signal=44%	Rps18l1/Syk/Rpl31/Rpl26/Il6r/Rpl10a
rno03010	Ribosome	DE signaling pathways(rat&human)	130	-0.524010368	-1.735561635	0.000921044	0.004131539	0.002839308	Activation in older cells	850	tags=40%, list=9%, signal=37%	Rpl31/Rpl26/Rpl10a/LOC102555453/
hsa04810	Regulation of actin cytoskeleton	DE signaling pathways(rat&human)	170	-0.451665303	-1.50642152	0.003208955	0.01803654	0.013569628	Activation in older cells	667	tags=24%, list=6%, signal=23%	ENAH/FGD3/ROCK1/DIAPH1/BRK1/[illegible]
hsa05020	Prion disease	DE signaling pathways(rat&human)	207	-0.380215274	-1.301497788	0.062600321	0.206188034	0.155123707	Activation in older cells	540	tags=11%, list=5%, signal=10%	SLC25A4/TUBB2A/EGR1/NCF4/TUBA[illegible]
rno05020	Prion disease	DE signaling pathways(rat&human)	195	0.435973277	1.306223916	0.038318912	0.112449892	0.077278672	Inhibition in older cells	1186	tags=39%, list=13%, signal=35%	Cav1/Uqcr10/Uqcrq/Ndufa4/Cox7c/[illegible]
hsa05171	Coronavirus disease - COVID-19	DE signaling pathways(rat&human)	179	0.402647132	1.456922673	0.006009694	0.029241198	0.021999351	Inhibition in older cells	998	tags=32%, list=8%, signal=30%	ISG15/RPS20/RPL13A/C3/RPL7/RPL2[illegible]
rno04810	Regulation of actin cytoskeleton	DE signaling pathways(rat&human)	144	0.546646062	1.57121202	0.001819187	0.007436421	0.005110514	Inhibition in older cells	898	tags=20%, list=10%, signal=18%	Myl9/Myh11/Itga8/Mylk/Vcl/Actn1/A
hsa03010	Ribosome	DE signaling pathways(rat&human)	124	0.500131761	1.738499114	0.000579785	0.004359872	0.00328011	Inhibition in older cells	2006	tags=46%, list=17%, signal=39%	RPS20/RPL13A/RPL7/RPL27A/RPL23/

Figure S9. Screenshots of the vascular aging pathways software in rats, monkeys and humans.
