# Supplementary material for: Cross‐species signaling pathways analysis inspire animal model selections for drug screening and target prediction in vascular aging diseases
Source: Evol Appl. 2024 Jun 10;17(6):e13708. doi: 10.1111/eva.13708 (PMC11164676; doi:10.1111/eva.13708)
Supplement: Supplementary file 2 — Table S1. Table S2. Table S3. Table S4. Table S5. [file EVA-17-e13708-s002.pdf]

Table S1 Advantages and disadvantages of diverse animal models for atherosclerosis.

Biological model		Advantages	Disadvantages	Reference
in vivo	Rodent	short reproductive cycle, relatively inexpensive	physiological structure differs significantly	(Leitersdorf et al., 2004; José J Fuster et al., 2012)
	Non-human primates	physiological structure is akin to humans	expensive, long reproductive cycle	(Godfrey S Getz et al., 2012; J Shim et al., 2016)
in vitro	Spheroids	reproducibility, cost-effectiveness	limited ability to replicate arterial layering	(Jun Chen et al., 2022)
	Cell-laden hydrogel structures	mimic human blood vessel wall layers	poor reproducibility, inadequate mechanical properties	(Chen CN et al., 2006)
	On-chip vasculature	provide diseased environment, achieve real-time imaging	no late-stage plaque research	(Mallone A et al., 2021)

Table S2 Overlapped signaling pathways in vascular aging from bulk-seq, GSE146972 and GSE164585 datasets.

Categories	Signaling pathways	NES="+"or"-"		
		Mice		
		Bulk-seq	GSE146972	GSE164585
SM	Cytokine-cytokine receptor interaction	+	+	+
SM	Chemokine signaling pathway	+	+	+
SM	Phagosome	+	+	+
SM	Cell adhesion molecules	+	+	+
SM	Neutrophil extracellular trap formation	+	+	+
SM	NOD-like receptor signaling pathway	+	+	+
SM	IL-17 signaling pathway	+	+	+
DE	Systemic lupus erythematosus	-	+	+

Note: SM, signaling pathways with consistent relative expression trends; DE, signaling pathways with opposite relative expression trends. NES= "+", Signaling pathways in a suppressed state during the vascular aging process. NES= "-", Signaling pathways in an activated state during the vascular aging process.

Table S3 Vascular sample information for rats, monkeys, and humans.

Species	GSE	Organisms	Tissues	Ages
Human	GSE216860	Homo spaiens	aorta tissues	the young group (3 months, 1 years, 3 years and 12 years)<br>the old group (57 years and 58 years)
Monkey	GSE117715	Macaca fascicularis	aorta tissues	the young group (4-6 years)<br>the old group (18-21 years)
Rat	GSE137869	Rattus norvegicus	aorta tissues	the young group (1 months)<br>the old group (23 months)

Table S4 Age reference table for rats, monkeys, and humans.

Species	Ages					
Human	0-10 years	10-20 years	20-30 years	38-47 years	56-69 years	70-90 years
Monkey	0-3 years	3-5 years	5-9 years	10-13 years	18-23 years	25-30 years
Rat	0-3 months	3-6 months	6-12 months	12-18 months	18-24 months	24-27 months

Table S5. Overlapped signaling pathways in vascular aging from GSE216860, GSE117715 and GSE137869 datasets.

Categories	Signaling pathways	NES="+"or"-"		
		rats	humans	monkeys
SM	Focal adhesion	-	-	-
SM	ECM-receptor interaction	-	-	-
SM	Platelet activation	-	-	-
SM	Relaxin signaling pathway	-	-	-
SM	AGE-RAGE signaling pathway	-	-	-
SM	Protein digestion and absorption	-	-	-
SM	Amoebiasis	-	-	-
SM	Proteoglycans in cancer	-	-	-
SM	Diabetic cardiomyopathy	+	+	+
SM	Antigen processing and presentation	+	+	+
DE	Oxidative phosphorylation	-	-	+
DE	Thermogenesis	-	-	+
DE	Lipid and atherosclerosis	+	+	-
DE	Chemokine signaling pathway	+	+	-
DE	IL-17 signaling pathway	+	+	-
DE	NF-kappa B signaling pathway	+	+	-
DE	Cellular senescence	+	+	-
DE	Toll-like receptor signaling pathway	+	+	-
DE	NOD-like receptor signaling pathway	+	+	-
DE	C-type lectin receptor signaling pathway	+	+	-
DE	TNF signaling pathway	+	+	-
DE	Alzheimer disease	-	-	+
DE	Parkinson disease	-	-	+
DE	Ribosome	+	-	+
DE	Osteoclast differentiation	+	+	-
DE	Alcoholic liver disease	+	+	-
DE	Huntington disease	-	-	+
DE	Pertussis	+	+	-
DE	Yersinia infection	+	+	-
DE	Chagas disease	+	+	-
DE	Influenza A	+	+	-
DE	Retrograde endocannabinoid signaling	-	-	+
DE	Viral protein interaction with cytokine and cytokine receptor	+	+	-
DE	Coronavirus disease-COVID-19	+	-	+

Note: SM, signaling pathways with consistent relative expression trends; DE, signaling pathways with opposite relative expression trends.
